# Elemental profiling reveals rhizosphere oxidation-mediated selective Fe exclusion mechanisms against Fe toxicity in rice in Madagascar

**DOI:** 10.1007/s11104-026-08919-4

**Published:** 2026-08-04

**Authors:** Toavintsoa Rajonandraina, Yoshiaki Ueda, Hanna Manwaring, Tovohery Rakotoson, Diarikanto Ny Avo Rafearivo, Takanori Okamoto, Tendro Radanielina, Andry Andriamananjara, Tantely Razafimbelo, Matthias Wissuwa, Guy J. D. Kirk

**Affiliations:** 1 Laboratory of Radioisotopes (LRI), University of Antananarivo, Antananarivo, Madagascar; 2 Crop, Livestock and Environment Division, Japan International Research Center for Agricultural Sciences (JIRCAS), Tsukuba, Ibaraki Japan; 3 Faculty of Engineering and Applied Sciences, Cranfield University, Cranfield, UK; 4 International Rice Research Institute (IRRI), Vietnam Office, Agricultural Genetics Institute, Hanoi, Vietnam; 5 Department of Plant Biology and Ecology, University of Antananarivo, Antananarivo, Madagascar; 6 PhenoRob Cluster and Institute of Crop Science and Resource Conservation (INRES), University of Bonn, Bonn, Germany

**Keywords:** Rice, Iron toxicity, Elemental profiling, Rhizosphere

## Abstract

**Background and aims:**

Iron (Fe) toxicity in lowland rice is caused by high Fe levels and mineral imbalances within plants. Thus, maintaining whole-plant mineral balance may be crucial for tolerance. However, there is limited evidence from field studies on elemental profiling that elucidates specific tolerance mechanisms. Here, we tested whether contrasting Fe tolerance mechanisms are associated with distinct patterns of nutrient uptake and distribution.

**Methods:**

We conducted multi-season experiments in Fe-toxic fields in Madagascar using rice genotypes with contrasting tolerance mechanisms. Elemental profiling was conducted for different parts of shoot tissues. Belowground Fe dynamics were investigated by measuring Fe plaque formation on roots and rhizosphere Fe^2^⁺ levels.

**Results:**

During the vegetative stages, tolerant genotypes that maintain low shoot Fe concentrations (excluders) showed a 48% reduction in shoot Fe uptake compared to other genotypes, while the uptake of other nutrients was less affected. Tolerant genotypes showed distinct within-plant nutrient allocation patterns. Excluders maintained lower Fe^2^⁺ concentrations in the rhizosphere soil solution, as well as high root:shoot Fe concentration ratios. A mathematical model of the effects of Fe oxidation on soil pH and cation exchange could explain the decreased uptakes of Fe and other cations by excluders.

**Conclusion:**

Genotypes with different tolerance levels and mechanisms exhibited distinct elemental profiles throughout growth. Genotypes with the same tolerance strategy may rely on distinct physiological mechanisms. Excluders selectively excluded Fe compared to other elements likely mediated by rhizosphere Fe oxidation.

**Supplementary Information:**

The online version contains supplementary material available at https://doi.org/10.1007/s11104-026-08919-4.

## Introduction

Rice (*Oryza sativa* L.) is a staple food for about half of the global population, and nearly three-quarters of its production is in irrigated lowlands, where continuous flooding is a common practice (Bouman et al. 2007; Muthayya et al. 2014). In these conditions, Fe^3^⁺ is reduced to soluble Fe^2^⁺, increasing iron (Fe) availability in soil solution and plant uptake. When it coincides with Fe-abundant soil, it leads to phytotoxic accumulation of Fe in plants and widespread Fe toxicity in lowland rice systems (Becker and Asch 2005). Fe toxicity typically causes foliar damage termed “leaf bronzing”, alongside reduced root growth and plant stunting, ultimately resulting in yield losses of 12%–100% (Howeler 1973; Sahrawat 2004).

Fe toxicity is associated with multifaceted plant nutrient disorders. Soils from Fe toxic fields typically contain suboptimal concentrations of most nutrients (Kirk et al. 2022) and deficiencies of phosphorus (P), potassium (K), magnesium (Mg), calcium (Ca), manganese (Mn) and zinc (Zn) are commonly found, either due to available concentrations in the soil or disruptions in uptake under Fe toxic conditions (dos Santos et al. 2020; Müller et al. 2015). Nutrient limitations can induce or exacerbate Fe toxicity. Potassium deficiency decreases root oxidizing power, Fe exclusion, root growth and antioxidant enzyme activity (Gao et al. 2014; Li et al. 2001). Accordingly, K supply under Fe toxicity conditions, which is partially mediated by a K channel AKT1, attenuates Fe uptake rates, tissue Fe accumulation, Fe toxicity-induced growth retardation and leaf bronzing (Wu et al. 2019). Likewise, Mg deficiency impairs vacuolar Fe storage and increases oxidative stress (Kobayashi et al. 2018). Accordingly, increased Mg supply, both under field and hydroponic conditions, confers tolerance through both inhibition of excess Fe uptake and increased tissue-level tolerance to high Fe (Rajonandraina et al. 2023b).

A similar pattern extends to P nutrition. Zheng et al. (2009) found that, under the same Fe supply level, P-deficient rice seedlings accumulated 2.1- and 1.8-fold more Fe in shoots and roots, respectively, than P-sufficient plants, accompanied by changes in the expression of Fe-responsive genes. Further, Zn deficiency reduces the activity of Cu/Zn superoxide dismutase, weakening antioxidant defenses and increasing Fe-induced oxidative stress (Obata et al. 1999). Consistent with this mechanism, field studies have shown that Zn supply can alleviate Fe toxicity. Across different cultivars and sites, Zn supply has been linked to a 20%–57% reduction in symptoms and a 36% increase in mean grain yield compared to control conditions (Sahrawat 2010; Shahid et al. 2014). These two-way interactions suggest that the severity of Fe toxicity is determined not solely by soil Fe concentrations but also by the occurrence of multiple nutrient disorders associated with or triggered by Fe toxic conditions.

Rice exhibits genetic variation in its tolerance to Fe toxicity (Matthus et al. 2015; Wu et al. 1997). Previous physiological studies have suggested four distinct mechanisms for tolerance (Audebert and Sahrawat 2000; Onaga et al. 2013; Rajonandraina et al. 2023a; Rosdianti et al. 2025; Stein et al. 2009, 2014): (i) Fe^2^⁺ exclusion at the root surface through root-induced oxidation forming insoluble ferric (Fe(III)) oxide in the rhizosphere and on root surfaces, (ii) Fe sequestration in roots to limit root-to-shoot translocation, (iii) Fe compartmentalization in organs with lower photosynthetic activity, such as stems, and (iv) detoxification of Fe-induced reactive oxygen species by antioxidant enzymes. The first two are referred to as “exclusion” mechanisms, whereby plants restrict aboveground Fe concentrations; the latter two are referred to as “inclusion” mechanisms, whereby plants tolerate large aboveground Fe concentrations. In a previous field investigation in the central highlands of Madagascar, we showed that rice genotypes with increased tolerance to Fe toxicity have distinct tolerance mechanisms, including both exclusion and inclusion processes (Rajonandraina et al. 2023a). Considering that Fe toxicity interacts strongly with imbalances of other nutrients, whole-plant nutrient dynamics may affect tolerance mechanisms under field conditions. However, the relationships between nutrient uptake patterns and Fe toxicity tolerance and genotypic variation in this are poorly understood.

Elemental profiling is a powerful approach to identify the physiological and genetic factors that regulate nutrient homeostasis (Huang and Salt 2016). Previous studies in rice have revealed both genotype- and environment-driven patterns of root nutrient uptake and root-to-shoot nutrient translocation and partitioning (Chengming et al. 2022; Du et al. 2020). In Fe toxicity studies conducted in both field and hydroponic conditions, elemental profiling has revealed the contribution of other minerals to determining tolerance (Sahrawat 2000; Olaleye et al. 2001; da Silveira et al. 2007). These studies highlight that the maintenance of elemental balance (e.g. P, K, Zn) constitutes an important aspect of tolerance mechanism, besides simply reducing Fe uptake. Whole-plant partitioning studies further show that such within-plant mineral distribution patterns in Fe toxicity conditions are under genetic regulations and changes during growth (Sperotto et al. 2012; Wu et al. 2019). However, one major limitation is that most elemental profiling datasets are based on whole tissue concentrations, and the potential impact of spatial partitioning that may influence symptoms and tolerance is not clearly understood. In addition, most studies only use one or two genotypes or hydroponic systems, with limited relevance to field conditions. Sampling is often limited to a single or two time points and primarily focuses on vegetative growth.

In this study, we evaluated the whole-plant multi-element profiles of seven rice genotypes with varying levels and types of tolerance to Fe toxicity in fields in the central highlands of Madagascar (Rajonandraina et al. 2023a). Our objective was to test whether contrasting Fe toxicity tolerance mechanisms are associated with distinct patterns of nutrient uptake and distribution. We hypothesized that (1) genotypes with contrasting tolerance mechanisms exhibit unique nutrient uptake and distribution patterns in Fe-toxic fields and these are shared among genotypes belonging to the same tolerance group; and (2) physiological mechanisms driving Fe exclusion alter rhizosphere nutrient uptake dynamics, shifting the overall plant nutrient patterns and distribution. We obtained elemental profiles in different tissues at multiple growth stages during a two-year field experiment and correlated them with plant phenotypes and Fe distribution patterns across genotypes.

## Materials and methods

### Experimental design

A field experiment was conducted at Sambaina, central highlands of Madagascar (18°53′12.77″S, 47°47′6.79″E) during the wet seasons of 2019–20 and 2020–21 (Years 1 and 2) as described in Rajonandraina et al. (2023a), which was later repeated for 2022–23 (Year 3) and 2024–25 (Year 4). Six lowland rice genotypes were used in Year 1, with a seventh genotype (Bahia) added in Year 2 (Table 1, Rajonandraina et al. 2023a). The soil at the site is a Gleysol with clay loam texture, pH 4.5, 62 g kg⁻^1^ organic carbon, 2.4 cmolc kg⁻^1^ cation exchange capacity and 57 g kg⁻^1^ total Fe, of which 10 g kg^−1^ is easily soluble upon reduction (Rakotoson et al. 2019). In Year 4, the same experiment was also conducted at Behenjy, central highlands of Madagascar (19°10′45.91″S, 47°29′38.90″E), as a non-Fe-toxic site under similar geographical and climatic conditions as Sambaina. The soil at Behenjy had a pH of 5.3, 45 g kg^–1^ organic carbon, and 26 g kg^–1^ total Fe.

**Table 1:** Origins, tolerances to Fe toxicity and reported tolerance mechanisms of rice genotypes used in this study

Full name	Short name	Origin	Mechanism	Tolerance
B14339-KA-28	KA-28	Indonesia	Includer	Tolerant
Bahia*	Bahia	Spain	Excluder	Tolerant
Ciherang	Ciherang	Indonesia	Includer	Sensitive
IR64	IR64	IRRI	Includer	Sensitive
NERICA-L-43	L43	AfricaRice	Excluder	Tolerant
Tsipala 421	Tsipala	Madagascar	Excluder	Tolerant
X265	X265	Madagascar	Includer	Tolerant

* Bahia was added in Year 2 only (2020–2021)

A completely randomized design with four replications was employed with 7 genotypes (Table 1). Subplots of a single genotype (2 m^2^ in Year 1; 1.6 m^2^ in Year 2) were randomly assigned within each replicate block and separated by bunds. Fertilizer was broadcast and incorporated at rates of 50 kg N ha⁻^1^ (urea), 20 kg P ha⁻^1^ (triple superphosphate) and 20 kg K ha⁻^1^ (potassium sulfate). Seeds were initially sown in a nursery and transplanted after 21 days. Before transplanting, the soil was irrigated from a nearby river, hand-plowed to a depth of 20 cm, then harrowed and leveled. Transplanting was done at 20 cm ×20 cm spacing with one seedling per hill. Fields were flooded to maintain at least 10 cm of standing water until the harvesting stage. Manual weeding was performed twice before the booting stage.

### Shoot sampling and analysis

Elemental profiling of shoot tissues was conducted during two consecutive cropping seasons (Year 1 and Year 2). Samples were collected at four growth stages: tillering, booting, flowering and maturity. At each stage, sampling included different tissues: the youngest leaf (YL), middle leaves (ML), old leaves (OL) and stem/leaf sheath (ST). Additionally, flag leaves (FL) were sampled at flowering and panicles (PN) at maturity. Two randomly selected hills per subplot were sampled and plants were immediately separated into component tissues in the field. Samples were stored in paper bags, oven-dried at 60 °C for 48 h and then weighed.

The dried tissues were ground using a mill (ZM200, Retsch, Haan, Germany) with 0.2 mm sieve. Concentrations of Fe, P, K, Ca, Mg, Na, Mn and Zn were determined for each tissue. The Fe concentration was measured using the micro-method described by Hartmann and Asch (2018). In brief, Fe was extracted from powdered, dried tissue using 500 mM sodium dithionite, followed by a colorimetric assay with 2,2’-dipyridyl, and quantified using a standard curve prepared with serially diluted Fe standard solutions. For the other nutrients, the powdered tissues were digested in concentrated HNO_3_ and H_2_O_2_ using a microwave digestion system (MARSXpress, CEM Corporation, Mathews, NC, USA). The resulting digests were analyzed using ICP-MS (NexION 350, PerkinElmer, Boston, MA, USA).

### Root and rhizosphere sampling and analysis

The analysis of Fe concentration in root tissue, root plaque and soil solutions was conducted in Year 4. Root samples were collected from an area of approximately 20 × 20 to 20 cm depth using a trowel. Initially, excess soil was removed from roots with field water, followed by thorough washing with tap water to remove any remaining soil residues. To remove Fe plaque, a representative subsample was immersed in 30 mL of dithionite-citrate-bicarbonate (DCB) solution consisting of 0.03 M sodium citrate (Na₃C₆H₅O₇0.2H₂O), 0.125 M sodium bicarbonate (NaHCO₃) and 0.115 M sodium dithionite (Na₂S₂O₄) for 2 h with occasional shaking (after Otte et al. 1989). After plaque removal, roots were rinsed twice with deionized water, oven-dried at 60 °C for 48 h and weighed. Approximately 50 mg of dried root tissue was then ground in 2.0 mL microtubes with two zirconia beads (AsOne, Japan) using a TissueLyser (Qiagen, Hilden, Germany) at a frequency of 25 Hz for 3 min. Iron concentrations in root tissue and extracted plaque were measured using the established micro-method of Hartmann and Asch (2018) as above.

Rhizosphere soil solution was collected at the booting stage using Rhizon samplers (Rhizosphere Research Products, Wageningen, Netherlands) inserted to a depth of 10 cm below the flooded soil surface. Samples were collected into pre-evacuated 12 mL glass vials and stored at 4 °C. Prior to Fe analysis, 100 mg of sodium dithionite was added to the solution to solubilize any Fe precipitate that may have formed during sample collection and storage. Fe concentrations were measured by the micro-method of Hartmann and Asch (2018).

### Gene expression

Leaf samples for gene expression analysis were collected in Year 3 at the flowering stage. The middle part of the flag leaf from a tiller at the grain filling stage was cut into 2–3 cm segments, washed with detergent to hydrophilize the surface, rinsed with tap water and treated with Sample Protector for RNA/DNA (Takara, Kusatsu, Japan) using a previously reported infiltration protocol (Ueda 2024). The samples were then stored at 4 °C for 24 h until RNA extraction.

After briefly rinsing the leaf segments with deionized water, RNA was extracted using the ISOSPIN Plant RNA kit (Nippon Gene, Tokyo, Japan) and cDNA was synthesized using the Prime-Script RT Master Mix reagent (Takara). Quantitative PCR was performed using the CFX96 Real-Time PCR Detection System (Bio-Rad, Hercules, CA, USA) with a reaction mixture containing Takara TB Green *Premix Ex Taq* II (Takara) and gene-specific primers (Table S1). Gene expression levels were quantified using the relative standard curve method, with *OsC3H38* (Os05g0564200) used as the internal reference gene.

### Parameters for modelling rhizosphere chemistry

Soil from the Sambaina field site was air dried and sieved to < 2 mm. Approximately 1.5 kg portions were mixed with 7.5 g of powdered rice straw in 3 L glass jars and saturated with sufficient deionized water to give 5 cm of standing water on the surface. Three Rhizon samplers (Rhizosphere Research Products) were placed vertically in the soil and the soil was left to reduce at 30˚C for 28 days. Periodically soil solution was withdrawn through the Rhizon samplers and pH, Eh and dissolved CO_2_ were measured. After 28 days, 5-cm^3^ of slurry were withdrawn by glass pipette into 35 cm^3^ centrifuge tubes containing 25 cm^3^ of 1 M NH_4_OAc at pH 2.8. The tubes were shaken for 30 min and Fe in the centrifuged, filtered extracts measured by ICP-MS. Further 5 cm^3^ portions were shaken for 4 h with 25 cm^3^ of deoxygenated 5 mM KCl containing 0–2.5 mM HCl, and the suspension pHs measured. The amount of acid added per unit mass of soil (Δ[HS], mmol kg^−1^) was calculated and the soil pH buffer power obtained from a plot Δ[HS] against pH.

### Data analysis

To eliminate inherent differences in nutrient demand among genotypes, nutrient-to-Fe concentration ratios (K:Fe, P:Fe, Mg:Fe, Ca:Fe, Na:Fe, Mn:Fe, and Zn:Fe), which may serve as a stoichiometric measure of competition between Fe and other cations (Howeler 1973), were calculated by dividing each nutrient concentration by the Fe concentration in the same sample, and interpreted alongside their corresponding absolute values.

Statistical analyses were performed using RStudio (Version 2025.05.0 + 496, Posit, Boston, USA), with a significance threshold set at *P* < 0.05. For normally distributed trait values, differences among genotypes and tolerance mechanisms were assessed using a mixed-model ANOVA implemented with the lmer function from the lme4 package (Bates et al. 2015). Genotypes or tolerance mechanisms were considered fixed effects, while year was treated as a random effect to account for inter-annual variability. When significant effects were observed, post-hoc comparisons were performed using Tukey’s Honest Significant Difference (HSD) test with the emmeans package (Lenth et al. 2021). For non-normally distributed trait values (shoot-to-root iron concentration ratio), a non-parametric Kruskal–Wallis test was adopted, followed by post-hoc comparisons by Dunn’s test.

## Results

**Fig. 1:**
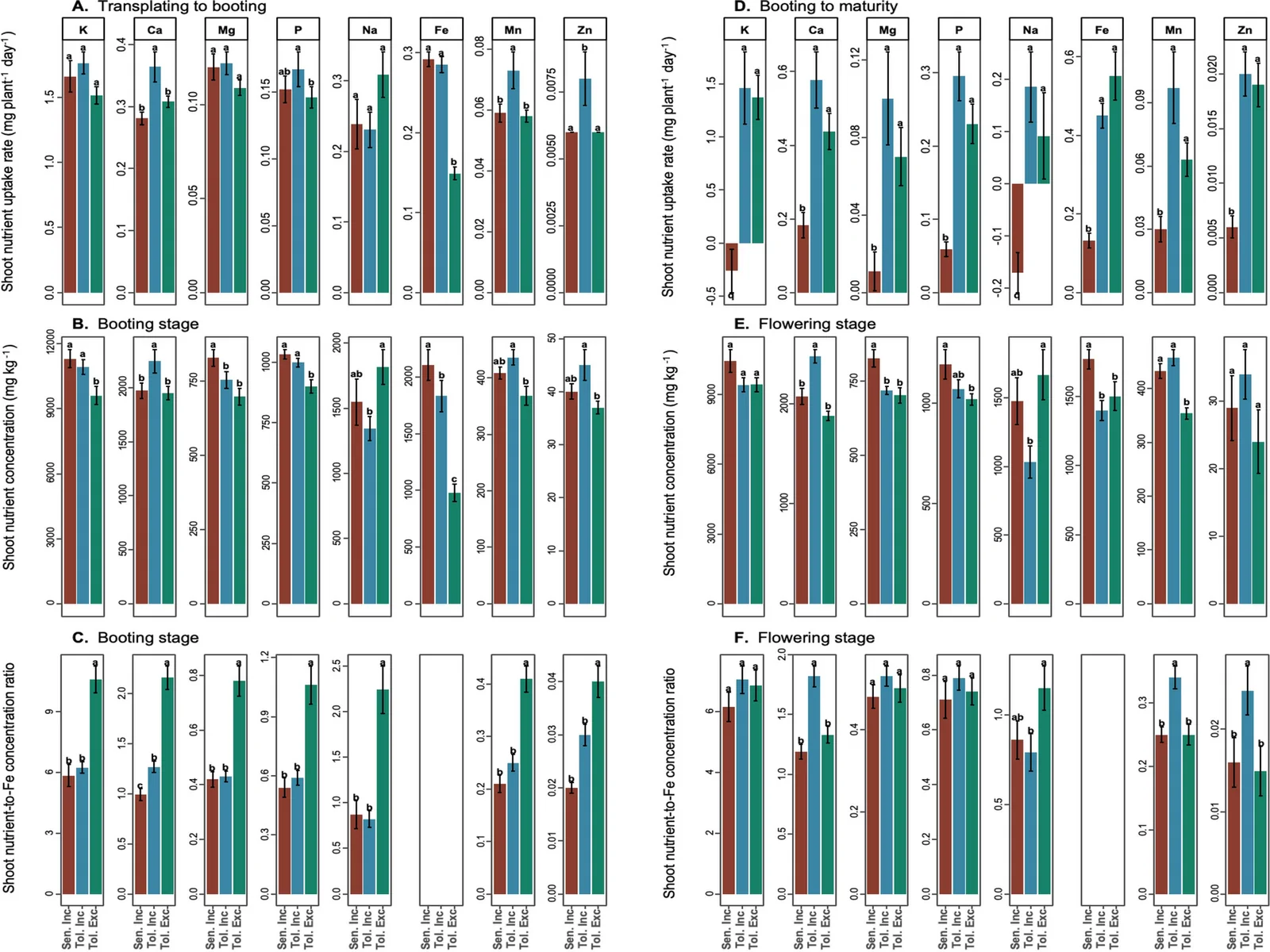
Plant nutritional status at whole shoot level for different tolerance mechanisms: **A**, **D** nutrient uptake rate; **B**, **E** nutrient concentration; **C**, **F** nutrient-to-Fe concentration ratio. **A**-**C** are from vegetative growth (A: transplanting to booting; B, C: booting stage). **D**-**F** are from reproductive growth (D: booting to maturity; E, F: flowering stage). Data are means ± standard errors across 2 years (*n* = 16 for includer, *n* = 20 for excluder). Common letters indicate no significant difference by Tukey’s HSD test

**Fig. 2:**
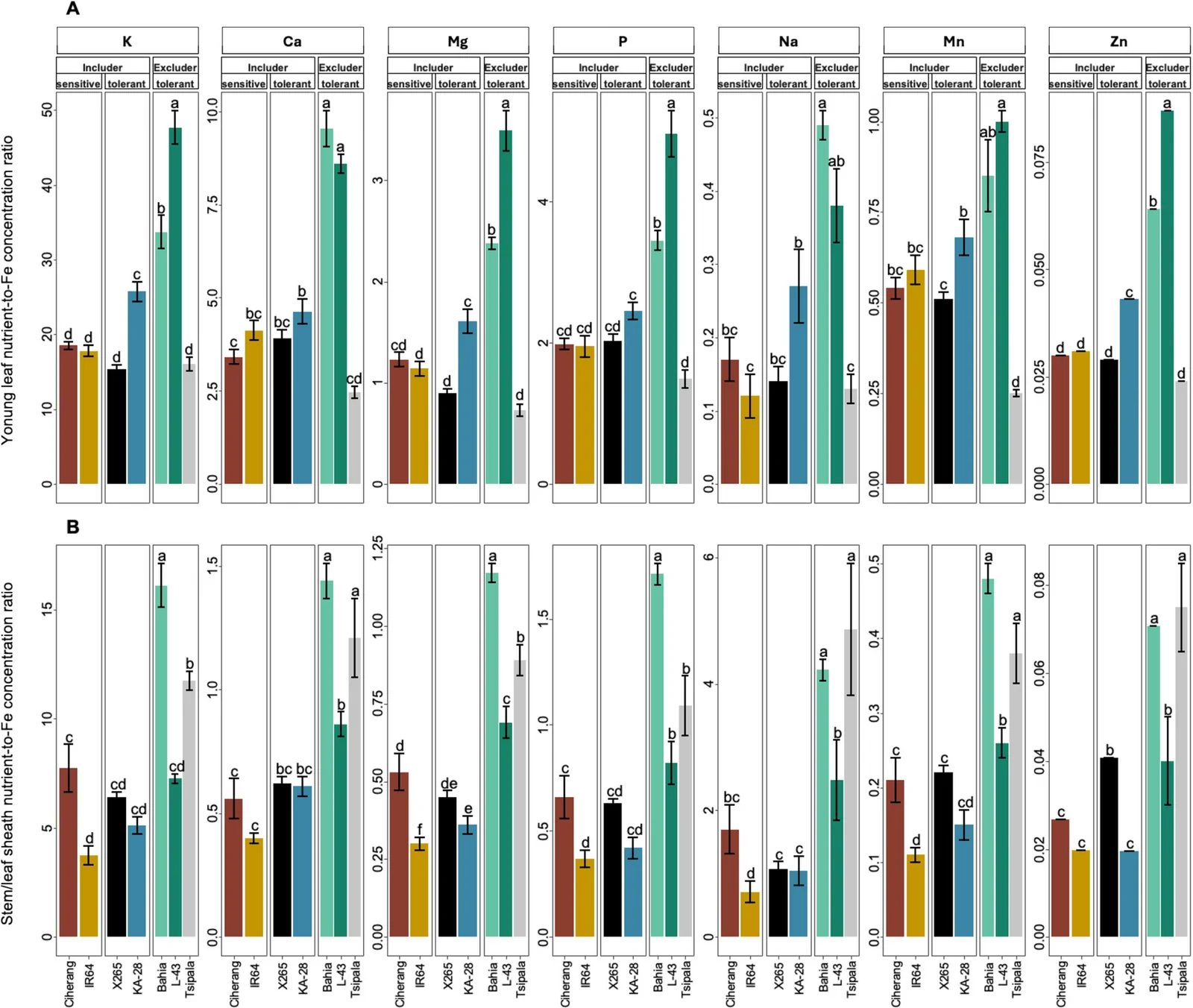
Nutrient-to-Fe concentration ratio in plant parts at booting stage: **A** young leaves; **B** stem/leaf sheath. Data are means ± standard errors across 2 years (*n* = 4 for Bahia; *n* = 8 for the other genotypes). Common letters indicate no significant difference by Tukey’s HSD test

**Fig. 3:**
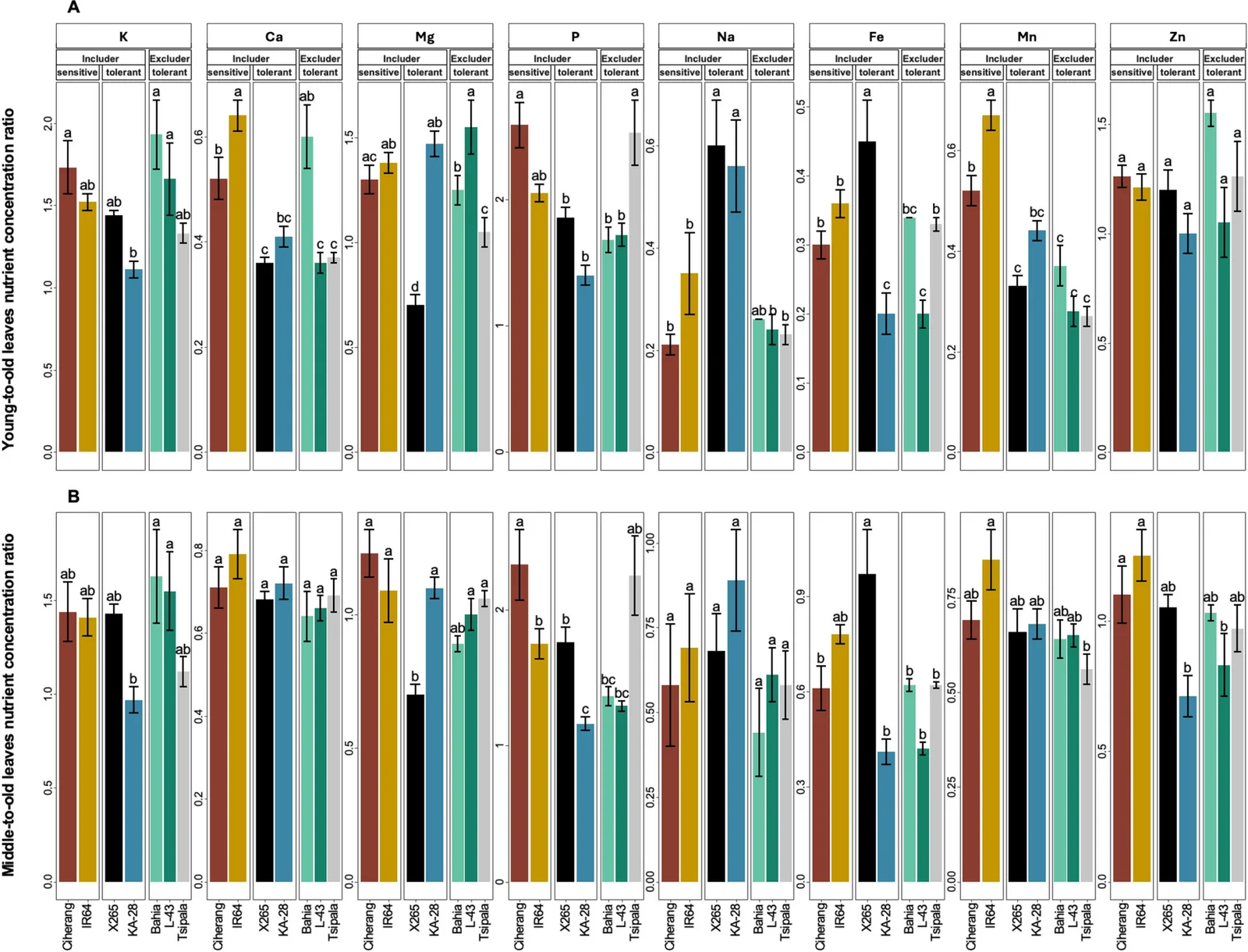
Nutrient concentration ratio between plant parts at booting stage: **A** young-to-old leaves (YL/OL), **B** middle-to-old leaves (ML/OL). Data are means ± standard errors across two years (*n* = 4 for Bahia; *n* = 8 for the other genotypes). Common letters indicate no significant difference by Tukey’s HSD test

**Fig. 4:**
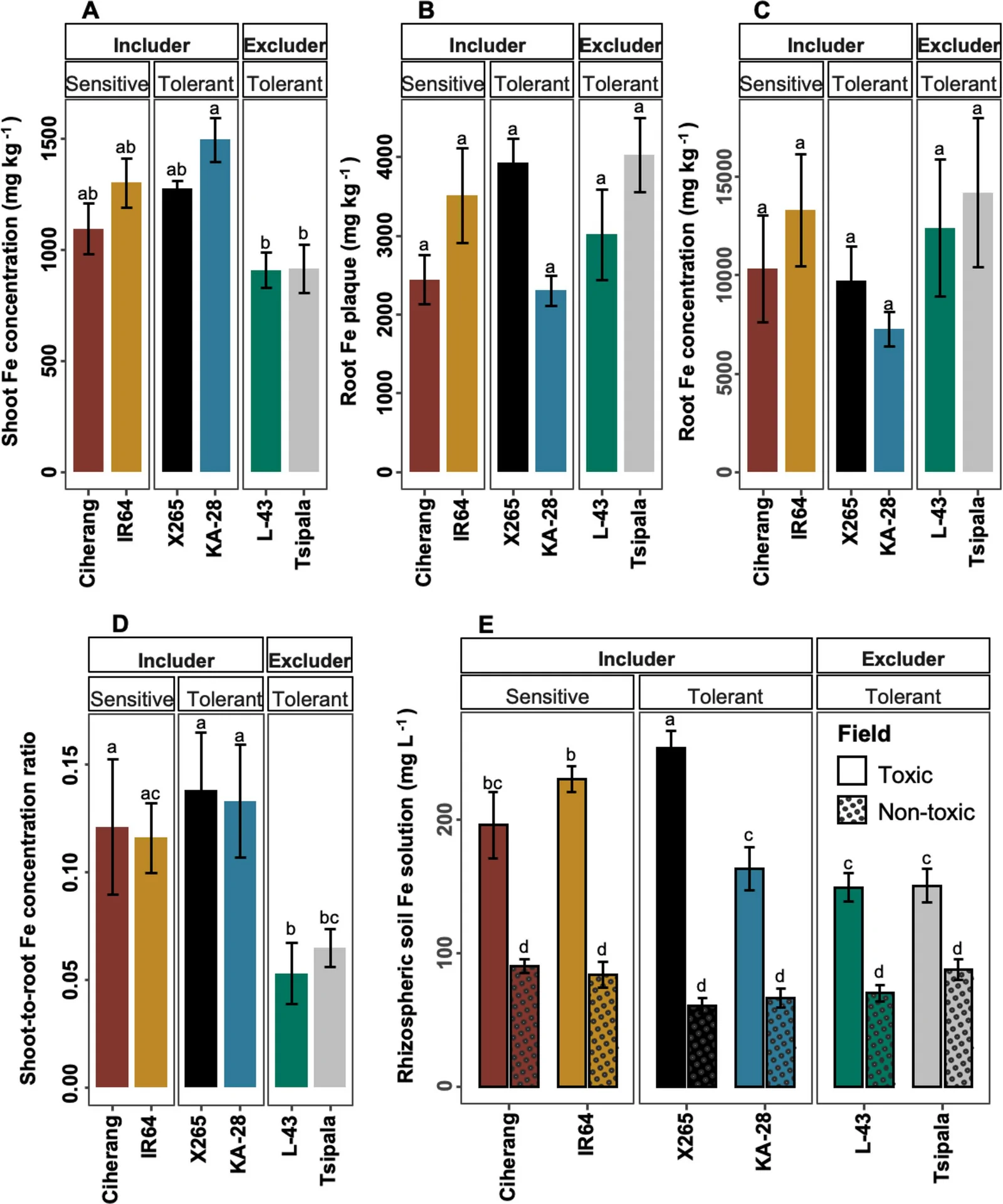
**A**-**C**, **E** Iron concentration at the booting stage in **A** shoot, **B** root plaque, **C** root, and **E** rhizospheric soil solution. **D** Shoot-to-root iron concentration ratio. **A**-**D** are from the toxic field, and **E** are from both toxic and non-toxic fields. Data are means ± standard errors (*n* = 4 for A-D; *n* = 6 for E). Common letters indicate no significant difference by Tukey’s HSD test (**A**-**C**, **E**) or Dunn’s test (**D**)

**Fig. 5:**
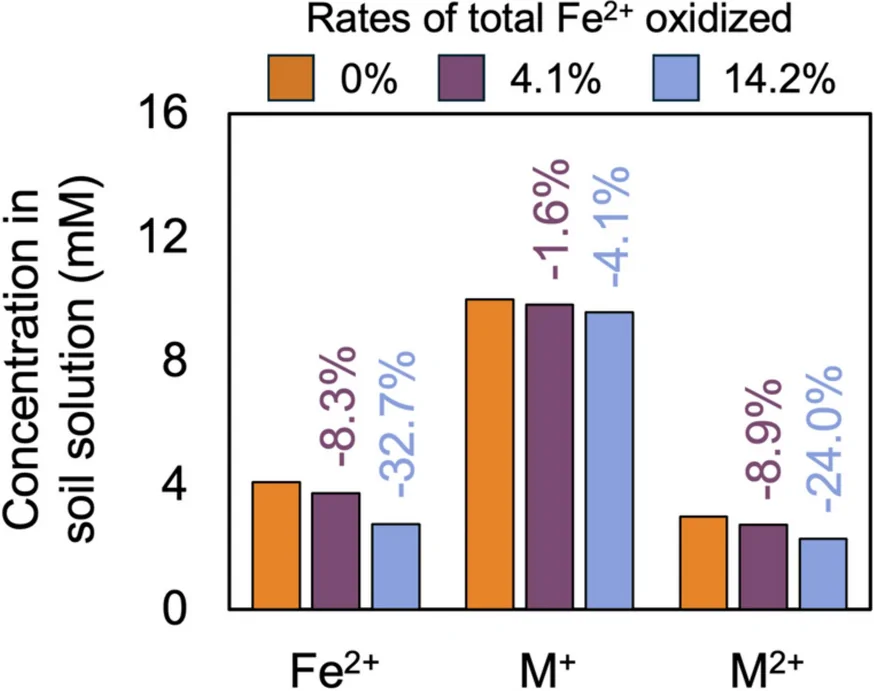
Calculated changes in concentrations of Fe^2+^, and monovalent (M^+^) and divalent (M^2+^) cations in soil solution upon oxidation of Fe. The percentage indicates changes from 0% oxidation

**Fig. 6:**
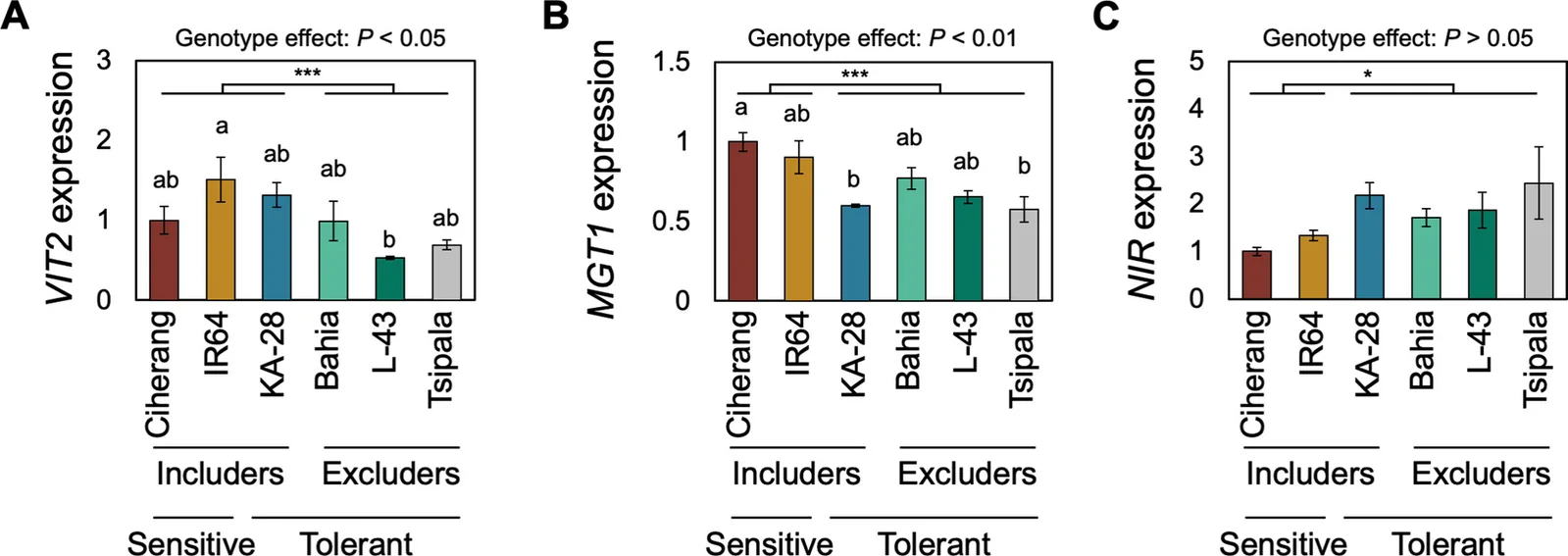
Gene expression analysis in flag leaves. **A** Expression of *VIT2* that showed significant difference between includers and excluders. **B**-**C** Expression of *MGT1* (**B**) and *NIR* (**C**). Data are means ± standard errors (*n* = 3). One-way ANOVA was conducted to analyze genotype effects, and the result is shown above each graph. Common letters indicate no significant difference in Tukey’s HSD test. The values of includers and excluders (**A**) or sensitive and tolerant accessions (**B**, **C**) were compared by two-tailed Student’s *t*-test, and the significance is shown with asterisks. *, *P* < 0.05; ***, *P* < 0.001

To systematically evaluate physiological responses to Fe toxicity, the data are presented in a targeted progression. We first describe whole-shoot dynamics across growth stages (Fig. 1), then focus on the booting stage by examining aboveground tissue-specific partitioning (Figs. 2 and 3) together with belowground Fe dynamics and rhizosphere chemistry (Figs. 4 and 5) and finally present the associated molecular responses (Fig. 6).

### Nutrient uptake patterns and ratios across the growth stages

The data were initially analyzed separately for each genotype (Fig. S1) and then grouped into sensitive includers (IR64, Ciherang; showing high shoot Fe concentration and susceptibility), tolerant includers (X265, KA-28; showing high shoot Fe concentrations and tolerance), and tolerant excluders (L-43, Tsipala, Bahia; showing low shoot Fe concentrations and tolerance) based on their established physiological characteristics in Fe toxic field conditions (Rajonandraina et al. 2023a; Table 1; Fig. 1). During vegetative growth, shoot uptake rates of Ca, P, Fe, Mn and Zn differed among the three groups, with tolerant includers consistently exhibiting the highest uptake rates. However, there were no differences in K, Mg and Na uptake rates (Fig. 1A). The most notable difference was in Fe uptake rates, with tolerant excluders having only about 48% of that of includers. Tolerant excluders also had lower (78%–87%) Ca, P, Mn and Zn uptake rates than tolerant includers, but comparable K, Mg and Na uptake rates.

At the booting stage, these nutrient uptake patterns were reflected in shoot concentrations, with significant differences among the groups in all analyzed nutrients (Fig. 1B; Table S2). Tolerant includers generally maintained the highest element concentrations, except for Na and Mg. In contrast, tolerant excluders had the lowest concentrations, except for Na which was notably higher. Excluders had lower (83%–90%) K, Ca, Mn, P and Zn concentrations than tolerant includers and substantially lower Fe concentrations (~53%). Nutrient-to-Fe concentration ratios at the booting stage reflected these patterns and differed among the three groups (Fig. 1C). Notably, tolerant excluders exhibited a higher nutrient-to-Fe concentration ratio (43%–62%) compared to both sensitive and tolerant includers. This suggests that the excluders specifically excluded Fe through selective Fe exclusion mechanisms that affect the uptake of other nutrients to a lesser extent. Conversely, includers maintained lower nutrient-to-Fe ratios overall, with tolerant includers showing a slightly higher Ca-to-Fe ratio compared to sensitive includers.

A marked shift in nutrient uptake occurred between vegetative and reproductive growth (Fig. 1D; Fig. S1). During reproductive growth, both tolerant groups (includers and excluders) consistently displayed higher uptake rates for all nutrients compared to sensitive includers. Sensitive includers exhibited significantly lower uptake rates, with reductions of 63%–87% for Ca, Fe, Mg, Mn, P and Zn compared to tolerant genotypes. These differences in reproductive uptake rates translated into significant differences in shoot Ca, Mg, P, Na, Fe and Mn concentrations at flowering, although no significant differences were observed for K and Zn (Fig. 1E). Sensitive includers exhibited the highest shoot concentrations despite their lower uptake rates, suggesting growth cessation and limited dilution effects. In contrast, tolerant genotypes had lower shoot nutrient concentrations during reproductive growth despite higher uptake rates.

At the flowering stage, significant differences among tolerance mechanisms were observed in Ca-, Na-, Mn- and Zn-to-Fe ratios but not for K-, Mg- and P-to-Fe ratios (Fig. 1F). The distinction between excluders and includers seen at the booting stage was no longer evident. Specifically, tolerant includers had higher Ca-, Mn- and Zn-to-Fe ratios, while tolerant excluders had higher Na-to-Fe ratio. These results suggest that the Fe exclusion mechanism may weaken or disappear during reproductive growth, affecting the nutrient balance of excluders.

### Nutrient distribution relative to Fe in plant tissues during vegetative growth

We investigated internal distribution patterns of Fe and other elements, focusing on the booting stage when tolerance mechanisms are expected to be active and differences among groups are most pronounced (Fig. 1) (Rajonandraina et al. 2023a). To examine nutrient distribution patterns relative to Fe concentrations, we calculated nutrient-to-Fe concentration ratios in young leaves and stem/leaf sheaths for each genotype (Fig. 2). Significant genotypic differences were observed in young leaves, with excluder genotypes (L-43 and Bahia) consistently showing the highest nutrient-to-Fe ratios, exceeding those of includers by 37%–60%. Interestingly, Tsipala, although classified as an excluder, had nutrient-to-Fe concentration ratios closer to includers, indicating a unique mechanism. Conversely, includer genotypes generally had low nutrient-to-Fe ratios, albeit with some internal variability. Specifically, KA-28 exhibited nutrient-to-Fe ratios 18%–47% higher than other includers, suggesting diversity within the includer group linked to internal nutrient distribution. This is mainly attributed to lower Fe concentrations in young leaves of KA-28, while concentrations of other minerals were comparable with other includers (Table S2). Similar trends were observed in middle leaves and old leaves, with two excluders (Bahia and L-43) consistently exhibiting high nutrient-to-Fe ratios across all positions of leaves (Fig. S2). The exception was the high Ca-, Mg-, P- and Mn-to-Fe ratios observed in old leaves of X265 compared to other includers.

Genotype-specific differences were evident also in stem/leaf sheaths (Fig. 2B). The excluder Tsipala displayed an opposite pattern to what was observed in young leaves, with significantly higher nutrient-to-Fe ratios across all measured nutrients, similar to another excluder, Bahia. Meanwhile, the other excluder, L-43, exhibited relatively lower nutrient-to-Fe ratios compared to Bahia and Tsipala but still surpassed includers for nutrients including Ca, Mg, P, Na and Mn. As with young leaves, includer genotypes consistently showed lower nutrient-to-Fe ratios in stem/leaf sheaths, despite internal variation within groups (Fig. 2B). These findings highlight that selective Fe exclusion during vegetative growth (Fig. 1A, B) leads to higher nutrient-to-Fe concentration ratios in excluder genotypes, suggesting a targeted nutritional balancing that favours other nutrients at the expense of Fe for excluders.

### Nutrient partitioning among different plant tissues during vegetative growth

Significant differences were observed in the young-to-old leaves nutrient concentration ratio across all nutrients, except for Zn (Fig. 3A). Tolerant includers commonly exhibited a lower ratio of K, P, Ca and Mn compared to sensitive includers, with respective decreases of 25%, 37%, 51% and 63%. However, Na was notably higher in both tolerant includers, averaging 74% greater than in sensitive includers. Despite both being tolerant includers, KA-28 and X265 differed distinctly in two key aspects. KA-28 exhibited a 53% higher Mg ratio, whereas X265 had a 55% higher Fe ratio compared to KA-28. No clear pattern emerged among tolerant excluders, as substantial variability was observed both within excluder groups and across different nutrients.

For the middle-to-old leaf nutrient concentration ratio (Fig. 3B), significant differences were observed for K, Mg, P, Fe, Mn and Zn, but not for Ca and Na. Sensitive includers consistently displayed the highest ratio for all significantly affected nutrients. Whereas KA-28 maintained a consistently low ratio of K, P, Fe and Zn, and a high ratio for Mg, similar to sensitive includers and 37% higher compared to X265. Conversely, X265 did not maintain its lower ratios seen in young-to-old leaves, instead exhibiting ratios closer to sensitive includers, except for a lower Mg ratio. Comparisons between young leaves-to-stem and middle leaves-to-stem showed large genotypic differences, with no consistent trend across genotype groups (Fig. S3).

### Rhizospheric Fe dynamics and root Fe accumulation during vegetative growth

The elemental profiling suggested that genotypes classified as tolerant excluders had a substantial within-group variation in traits such as within-plant Fe partitioning but commonly had high element-to-Fe ratios. We reasoned that such selective shoot Fe exclusion is due to either 1) limited root-to-shoot Fe translocation or 2) decreased Fe uptake from the rhizosphere. To investigate this, we conducted another field experiment (Year 4), focusing specifically on belowground Fe dynamics. We confirmed that the plants behaved in a similar manner to previous field experiments in which elemental profiling data were obtained (Fig. 4A) (Rajonandraina et al. 2023a). Excluder genotypes exhibited the lowest shoot Fe concentration, with an average value of 912 mg kg^−1^, compared to a 41% higher in the includer genotypes, with KA-28 having the highest Fe concentration at 1494 mg kg^−1^.

The formation of root plaque is suggested as a mechanism to limit excessive Fe uptake in Fe toxic soils (Green and Etherington 1977). However, we found no significant differences in plaque concentrations among genotypes (Fig. 4B). Investigation from another field season (2023–24) showed no significant difference either (Fig. S4). Similarly, root Fe concentrations were not significantly different (Fig. 4C). However, shoot:root Fe concentration ratio was significantly lower for excluders, except for IR64 vs Tsipala comparison (Fig. 4D).

We then examined Fe concentrations in the rhizosphere soil solution (Fig. 4E). In the Fe toxic field, there were significant differences among genotypes, with tolerant excluders consistently having smaller Fe^2^⁺ concentrations, with an average reduction of 29% compared to includers. In contrast, includers X265, IR64 and, to a lesser extent, Ciherang had greater rhizosphere Fe^2^⁺ concentrations, while KA-28 showed values comparable to excluders, suggesting potential variation in mechanisms among includer genotypes. We conducted a similar analysis in a non-toxic field to determine if this effect was specific to the Fe toxic field. In the non-toxic field, rhizospheric Fe^2^⁺ concentrations ranged from 60 to 90 mg L^−1^, with no significant differences among genotypes, indicating that the reduction of rhizosphere Fe by excluders (L-43 and Tsipala) and KA-28 is a trait specific to Fe toxicity.

### Simulation of rhizosphere chemistry

We simulated the effects of Fe^2+^ oxidation on ion-dynamics in the rhizosphere using a model of cation exchange and pH changes with parameter values measured or estimated for the experimental soil (Note S1). The measured Fe^2+^ concentration in the soil solution was 4.2 mM when the soil was completely reduced and this decreased to 3.8 and 2.7 mM on average in the rhizosphere soil solution of includers and excluders, respectively (Fig. 4D). According to the model, these concentrations would be achieved when 3.9% and 14.2% of the total Fe^2+^ is oxidized. The modeling also suggests that the concentration of divalent cations in the rhizosphere solution would decrease to a greater extent than that of monovalent cations due to Fe^2+^ oxidation: oxidation of 3.9% of the Fe^2+^ would lead to a decrease in divalent cations in by 8.7% and 1.6%. Similarly, oxidation of 14.2% of the Fe^2+^ would cause divalents to decrease by 24.0% and 4.1% (Fig. 5).

### Molecular responses associated with the elemental profiling pattern

We analyzed the expression of nutrient-responsive genes to gain insights into genotypic differences in nutrient utilization patterns at the molecular level. To avoid potential biases from varying growth stages and climatic conditions, flag leaves from flowering tillers (Fig. S5) were collected on the same day. X265 was excluded from the analysis due to the unavailability of such a developmentally adjusted sample. The expression of *VIT2*, which encodes a vacuolar Fe transporter (Zhang et al. 2012), is strongly induced by Fe toxicity in leaves (Rosdianti et al. 2025; Aung et al. 2018). Significant genotypic differences were observed in the expression level of *VIT2*, with the includers (3 genotypes included) having > twofold higher expression than the excluders (3 genotypes included) (Fig. 6A).

MGT1 is a major Mg transporter responsible for around 30%–40% of Mg uptake (Chen et al. 2012), and its expression is induced by Fe toxicity in both shoot and root (Rajonandraina et al. 2023b). Reflecting the higher Mg concentrations, the expression of *MGT1* was significantly higher in sensitive includers (Ciherang and IR64) (Fig. 6B).

We also investigated nitrite reductase-encoding gene, *NIR*, which is critical for nitrogen (N) use efficiency (Yu et al. 2020). Despite the absence of significant genotypic differences when comparing 6 genotypes, the expression levels were significantly different between sensitive includers and tolerant groups, with the latter showing > 80% higher expression on average (Fig. 6C).

## Discussion

### Potential contributions of rhizosphere oxidation to observed differences in elemental profiling

Our previous study (Rajonandraina et al. 2023a) and Year 4 experiment in the current study consistently showed that shoot Fe concentrations of excluders were half of includers during vegetative growth. By contrast, shoot concentrations of Ca, P, Mn and Zn were only 10%–17% lower in excluders than includers. Together with the 29% lower rhizosphere Fe^2^⁺ concentration, this supports a model in which tolerant excluders primarily mitigate Fe toxicity by selectively excluding Fe^2^⁺ availability in the rhizosphere, thereby limiting root uptake and reducing the net translocation to shoots, although root retention may also play a role to some extent (discussed later).

At the vegetative stage, rates of P, K, Ca, Mn and Zn uptake were smaller in tolerant excluders than includers (differences were not significant for K and Mg), though not by as much as the differences in rates of Fe uptake. Shoot concentrations of all nutrients except Na were smaller in excluders than includers at the booting stage, and nutrient:Fe ratios were also smaller. Shoot concentrations of P, K and Mg were at or below thresholds for deficiency (1,000, 15,000 and 1,300 mg kg^−1^ respectively; Dobermann and Fairhurst 2000), but Ca, Mn and Zn concentrations were above thresholds (1,500, 40, 10 mg kg^−1^ respectively). Shoot Na concentrations were unusually high: Na/Ca ratios are typically 5–25 (Dobermann and Fairhurst 2000).

Differences in Fe uptake into roots may imply differences in oxidation of soluble ferrous iron (Fe^2+^ ions) to insoluble ferric iron (Fe(OH)_3_) in the rhizosphere. This occurs by release of O_2_ from roots after diffusing down through the roots’ internal gas channels (Becker and Asch 2005; Kirk et al. 2022). Upon oxidation of Fe^2+^ to Fe(OH)_3_ near the root surface, more Fe^2+^ diffuses towards the root from the bulk soil and is oxidized, resulting in an accumulation of Fe(OH)_3_ over a zone around the root. We found no differences among genotypes in Fe plaque on roots during two consecutive seasons, which does not support the hypothesis that Fe plaque plays an inhibitory role for Fe uptake in excluders at least at the booting stage under our experimental conditions. Nevertheless, Fe plaque formation is thought to reflect the degree of root-mediated O_2_ release and is more prominent during the early vegetative stage of rice roots (Nanzyo et al. 2010) and it may have functioned as a barrier at this growth stage in our experiments as well. It is conceivable that Fe plaque on the root surface was saturated and much of the Fe was oxidized at a distance from the root surface, as suggested previously (Begg et al. 1994). This is supported by the differences in Fe^2+^ concentrations in the rhizosphere soil solution despite the absence of difference in Fe plaque formation between genotypes.

### Potential mechanisms for genotypic differences in rhizosphere oxidation

The plant-availability of nutrient ions in the rhizosphere is sensitive to changes in soil chemistry induced by roots. These particularly include (1) Fe^2+^ oxidation to insoluble Fe(OH)_3_ and H^+^ ions (4Fe^2+^ + O_2_ + 10H_2_O = 4Fe(OH)_3_ + 8H^+^), tending to lower the rhizosphere pH; (2) release of H^+^ ions from roots to balance excess intake of cations (especially of NH_4_^+^ in anoxic flooded soil) over anions, also tending to lower the pH; (3) venting of soil CO_2_ through the roots, so depleting dissolved carbonic acid (H_2_CO_3_) in the soil solution and thereby offsetting some of the pH decrease (Kirk et al. 2022). Formation of Fe(OH)_3_ and acidification of the rhizosphere will together tend to decrease the plant-availabilities of both P and nutrient cations but differently for monovalent and divalent ones through mechanisms discussed by Kirk et al. (2022) (Note S1). In brief, as Fe^2^⁺ oxidizes, the rhizosphere pH and HCO₃⁻ concentration decrease, thereby lowering the overall strength of the soil solution. As a result, the concentrations of balancing cations in the solution must also decrease. The effect is greater for divalent than monovalent cations in accordance with the ratio law.

The modeling showed that around 14% of total Fe^2+^ is expected to be oxidized in excluders, and this would cause substantial decreases in concentrations of Fe^2+^ (−33%) and divalent cations (−24%) in the soil solution, but a much smaller decrease in monovalent cations (−4%) (Fig. 5). This could explain why the uptake rates of divalent cations (especially Ca^2^⁺, Zn^2^⁺, and Mn^2^⁺) were generally smaller in excluders than includers and to a greater extent than for monovalent cations (Fig. 1). Mg^2+^ uptake rates were also smaller, but to a lesser extent for reasons not explained by our model. We did not allow for the additional rhizosphere acidification due to the plant’s cation–anion balance, which may be comparable to that due to Fe^2+^ oxidation (Begg et al. 1994). That would further decrease cation concentrations in solution.

The main form of plant-available N in anoxic flooded soil is ammonium ions (NH_4_^+^). However, nitrate (NO_3_^−^) may be formed in the oxic rhizosphere by nitrification of NH_4_^+^. Our finding of greater expression of *NIR*, which is induced by nitrate-activated transcription factor NLP4 (Yu et al. 2020), in excluder genotypes (Tsipala, L-43) and KA-28 suggests a preference for NO_3_^−^. This may be significant because plant growth is generally improved when plants absorb their N as a mixture of NO_3_^−^ and NH_4_^+^ (Kronzucker et al. 2000). However, competition with Fe^2+^ oxidation for root-released O_2_ would mean nitrification would be at the expense of Fe exclusion. We also note that the net acid–base change in the rhizosphere will be the same with NO_3_^−^ uptake because, although the root exports two mol less H^+^ for each mol of NO_3_^−^ replacing a mol of NH_4_^+^, two mol of H^+^ are formed per mol of NH_4_^+^ nitrified. Hence, root-induced pH changes and their consequences for rhizosphere chemistry would be the same.

Greater O_2_ release from roots and genotype-specific exudation could potentially enrich Fe-oxidizing bacteria and nitrate-dependent Fe^2^⁺ oxidizers, resulting in lower Fe^2^⁺ levels (Chan et al. 2023; Oshiki et al. 2013; Watanabe et al. 2023). Excluders may avoid taking up Fe^2^⁺ by creating conditions in the rhizosphere that favour Fe^2^⁺-oxidizing bacteria such as the family *Gallionellaceae* (Okamoto et al. 2025), rather than relying solely on abiotic Fe(II) oxidation. Such microbe-mediated mechanisms align with growth stage-dependent changes in root exudate composition and microbe community structure (Fu et al. 2011; Li et al. 2017; Liu et al. 2025), with more assimilates directed towards reproductive organs during the reproductive stage. While this remains speculative in the context of the present study, further research focusing on the microbial composition of rhizosphere soils among includers and excluders would be valuable.

### Tissue-level exclusion

In addition to rhizosphere level exclusion, reduced root-to-shoot Fe translocation (root Fe retention) may also play a role, considering the higher root:shoot Fe concentration ratios in excluders (0.059 on average) than in includers (0.127 on average) (Fig. 4D). Because most Fe in xylem exists as an Fe-citrate complex, genotypic differences in this retention capacity may be driven by the expression patterns of xylem citrate transporter *FRDL1*, of which knockdown leads to reduced root-to-shoot Fe translocation (Yokosho et al. 2016; Ueda 2025).

At booting, excluders L-43 and Bahia showed higher nutrient:Fe ratios in young leaves than includers, indicating a selective restriction of Fe allocation to photosynthetic tissues to preserve nutritional balance. This implies a tissue-level Fe exclusion layered on top of plant-level exclusion. This selectivity could be partly mediated by the basal node, which modulates inter-organ nutrient allocation (Yamaji et al. 2013, 2024) through node-localized transporters such as Fe^2+^ efflux transporter IET1 (Che et al. 2025) and xylem citrate transporter FRDL1 (Ueda 2025). Investigations on whether such genes are important for characteristic Fe distribution in tolerant genotypes in the field would be of future interest.

Tsipala, another excluder, showed a unique profile, with low leaf nutrient:Fe ratios but with high stem/leaf sheath nutrient:Fe ratios. This suggests weaker node-mediated retention in Tsipala compared to L-43 and Bahia. Alternatively, this could be attributed to the tall phenotype of Tsipala compared to other excluders (Fig. S6), possibly mediated by gibberellin oxidase GA20ox2 (SD1). Previous studies showed that plants with functional SD1 have improved P and N utilization compared to those with non-functional types, affecting tissue element concentrations and uptake efficiency (Li et al. 2018; Zhu et al. 2025). These findings further indicate that genotypes may rely on distinct or partially independent physiological or molecular mechanisms, even within the same tolerance group. It is tempting to investigate if SD1 plays an additional role in Fe toxicity tolerance and within-plant Fe distribution, in addition to the rhizosphere-based mechanism in Tsipala.

### Genotype-specific nutrient utilization strategies related to tolerance

Tolerant genotypes, both includers and excluders, showed a 74% higher young-to-old leaf Na ratio than sensitive genotypes, indicating greater Na accumulation in young leaves. Since Fe toxicity can lead to secondary water stress and disrupt osmotic balance, this pattern suggests that Na may assist in osmotic adjustment in growing tissues. A previous study on African rice (*O. glaberrima*) suggested that the tolerant excluder accession TOG 7105 reduced osmotic potential under Fe toxicity, while the sensitive accession IRGC 104047 increased it (Majerus et al. 2007), highlighting the potential role of osmolytes in Fe exclusion. Studies on salinity stress show that vacuolar Na^+^ can serve as a more energy-efficient osmolyte than organic solutes (Chakraborty et al. 2020; Munns et al. 2016). Thus, it is possible that tolerant genotypes sequester Na^+^ in young leaf vacuoles to maintain turgor and ion balance while reducing reliance on organic osmolytes.

Among tolerant includers, KA-28 and X265 exhibited differences in nutrient allocation: KA-28 had a 53% higher Mg young-to-old ratio, while X265 had a 55% higher Fe young-to-old ratio. The lower Fe concentrations in young leaves of KA-28, with preferential Fe accumulation in older leaves, suggests Fe compartmentalization with potential support from Mg. These findings align with Mg supply alleviation of Fe toxicity and impaired vacuolar Fe sequestration under Mg deficiency (Kobayashi et al. 2018; Rajonandraina et al. 2023b), which is particularly beneficial in young leaves (Peng et al. 2019). By contrast, X265 had higher Fe levels in young leaves, suggesting a greater reliance on true tissue tolerance. Although these interpretations are based on correlations, the divergence indicates that genotypes with similar tolerance mechanisms can achieve tolerance through distinct nutrient allocation strategies. These observations highlight that investigations on individual genotypes are needed in the future to identify unique mechanisms carried by each tolerant genotype.

## Conclusions

Our elemental profiling study of rice genotypes with contrasting levels and mechanisms of tolerance to Fe toxicity showed that:

1. Excluder genotypes selectively exclude Fe with only minor reductions in Ca, Mn, Zn uptake.
2. Selective Fe exclusion is associated with lower rhizosphere Fe^2+^, achieved through root-driven Fe oxidation.
3. Genotypes employing the same tolerance mechanism may rely on distinct genetic and physiological factors to express their tolerance.

## Supplementary Information

Below is the link to the electronic supplementary material.

ESM 1 Supplementary Material 1 (PDF 1.09 MB)

ESM 2 Supplementary Material 2 (XLSX 16.4 KB)

## Data Availability

All data generated or analysed during this study are included in this published article and its supplementary information files.

## Abbreviations

FL: Flag leaf
LBS: Leaf bronzing score
ML: Middle leaf
OL: Old leaf
PN: Panicle
ST: Stem and leaf sheaths
YL: Young leaf
