# Supplementary material for: Elemental profiling reveals rhizosphere oxidation-mediated selective Fe exclusion mechanisms against Fe toxicity in rice in Madagascar
Source: Plant Soil. 2026 Aug 4;526(2):1533–49. doi: 10.1007/s11104-026-08919-4 (PMC13638738; doi:10.1007/s11104-026-08919-4)
Supplement: Supplementary file 1 [file 11104_2026_8919_MOESM1_ESM.pdf]

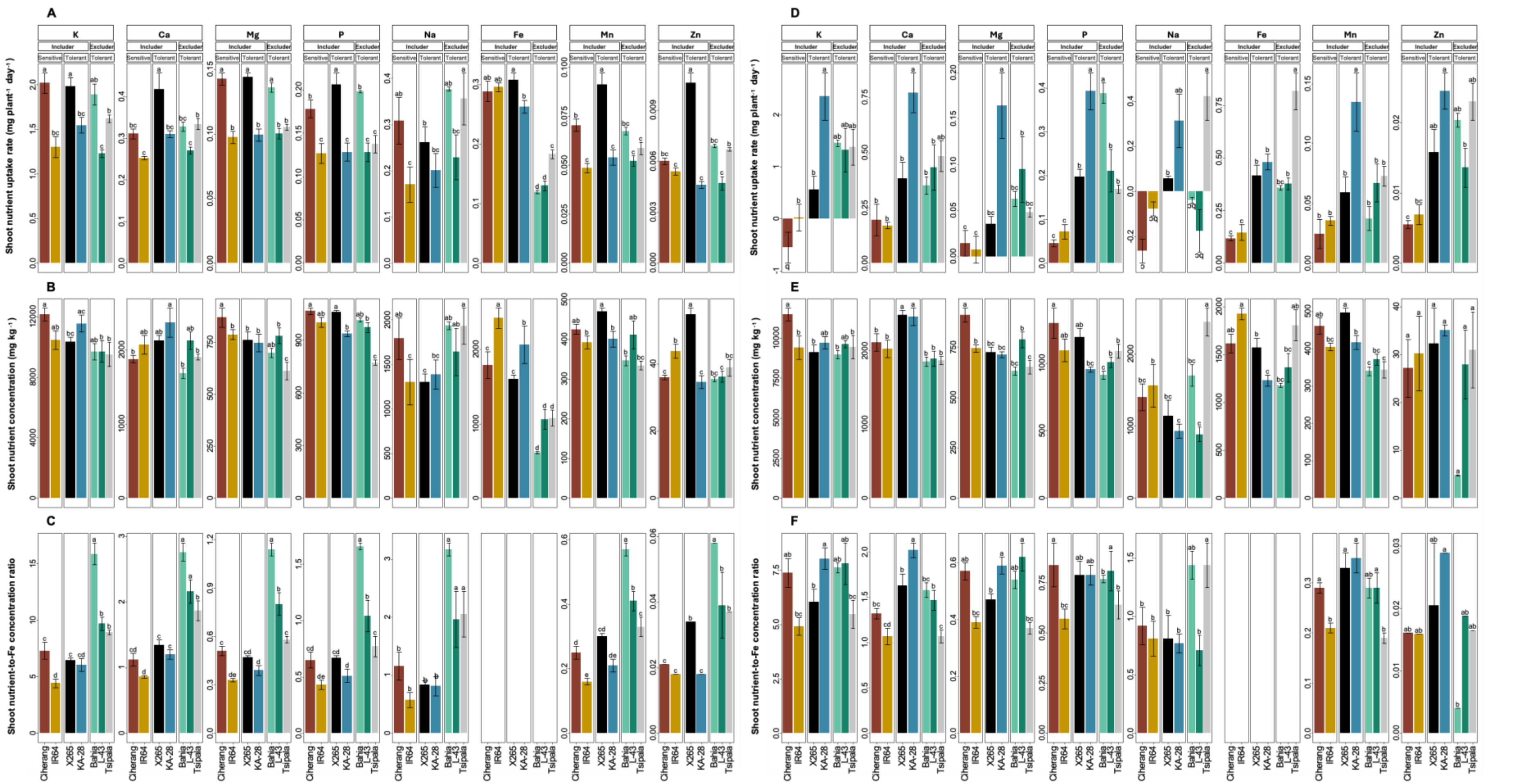

Fig. S1: Plant nutritional status at the whole shoot level for different genotypes.

(A, D) nutrient uptake rate; (B, E) nutrient concentration; (C, F) nutrient-to-Fe concentration ratio. (A-C) are from vegetative growth (A: transplanting to booting; B, C: booting stage). (D-F) are from reproductive growth (D: booting to maturity; E, F: flowering stage). Data are means ± standard errors (n = 4 for Bahia; n = 8 for the other genotypes). Common letters indicate no significant difference in Tukey's HSD test.

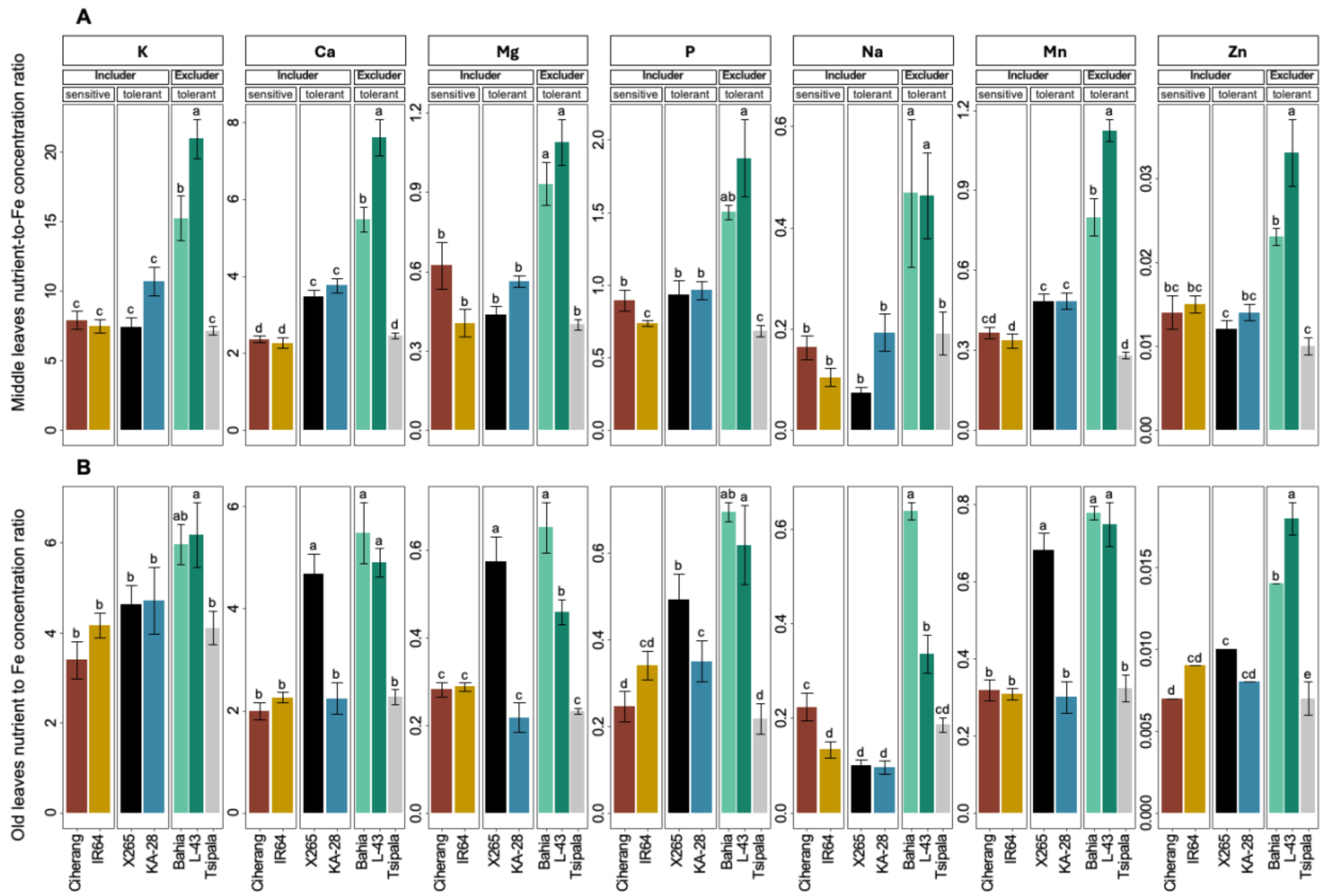

Fig. S2: Nutrient-to-Fe concentration ratio in plant parts at booting stage.

(A) Middle leaves; (B) Old leaves. Data are means ± standard errors (n = 4 for Bahia; n = 8 for the other genotypes). Common letters indicate no significant difference in Tukey's HSD test.

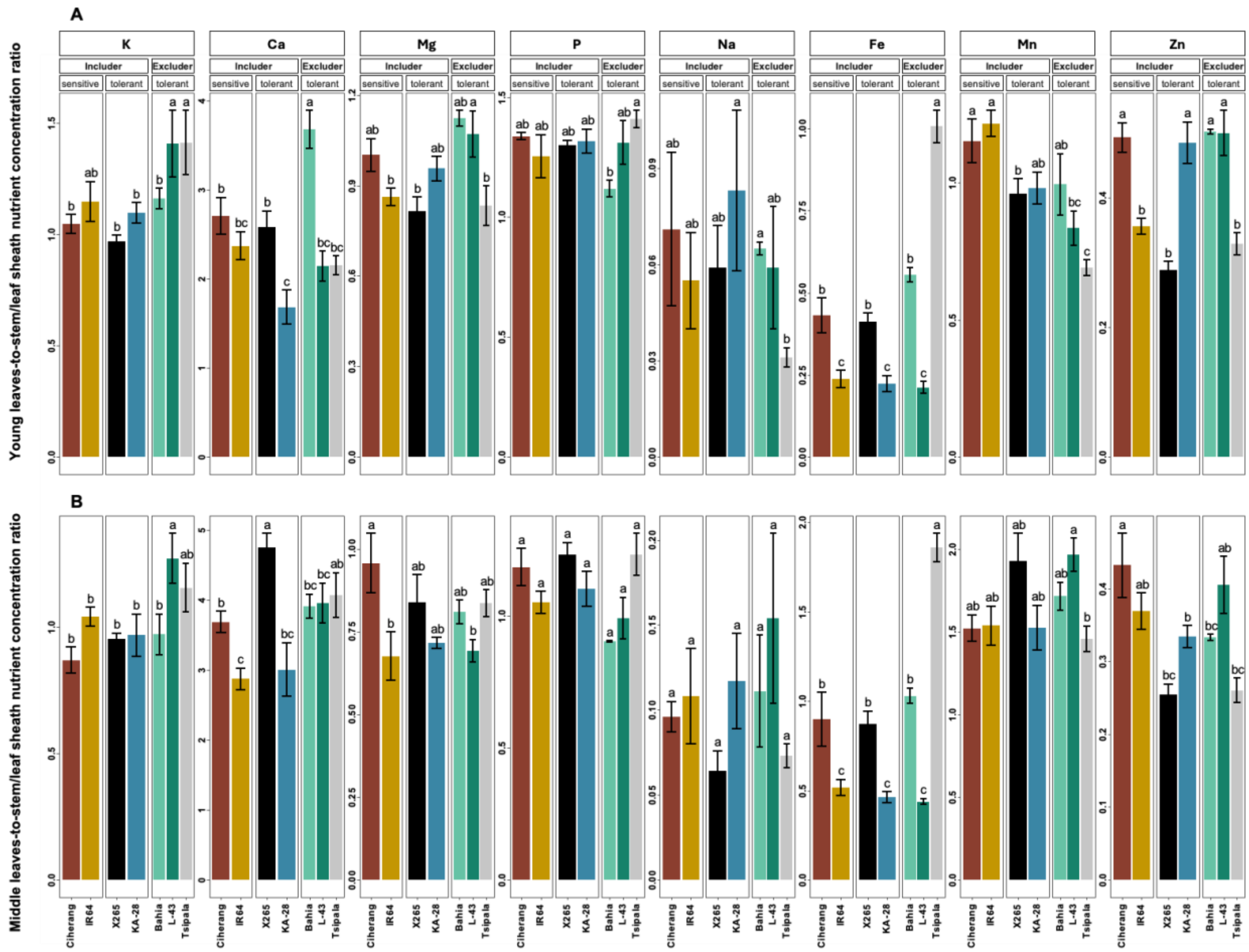

Fig. S3: Nutrient concentration ratios between plant parts.

(A) Young-to-stem/leaf sheath (YL/ST), (B) Middle-to-stem/leaf sheath (ML/ST). Data are means ± standard errors (n = 4 for Bahia; n = 8 for the other genotypes). Common letters indicate no significant difference in Tukey's HSD test.

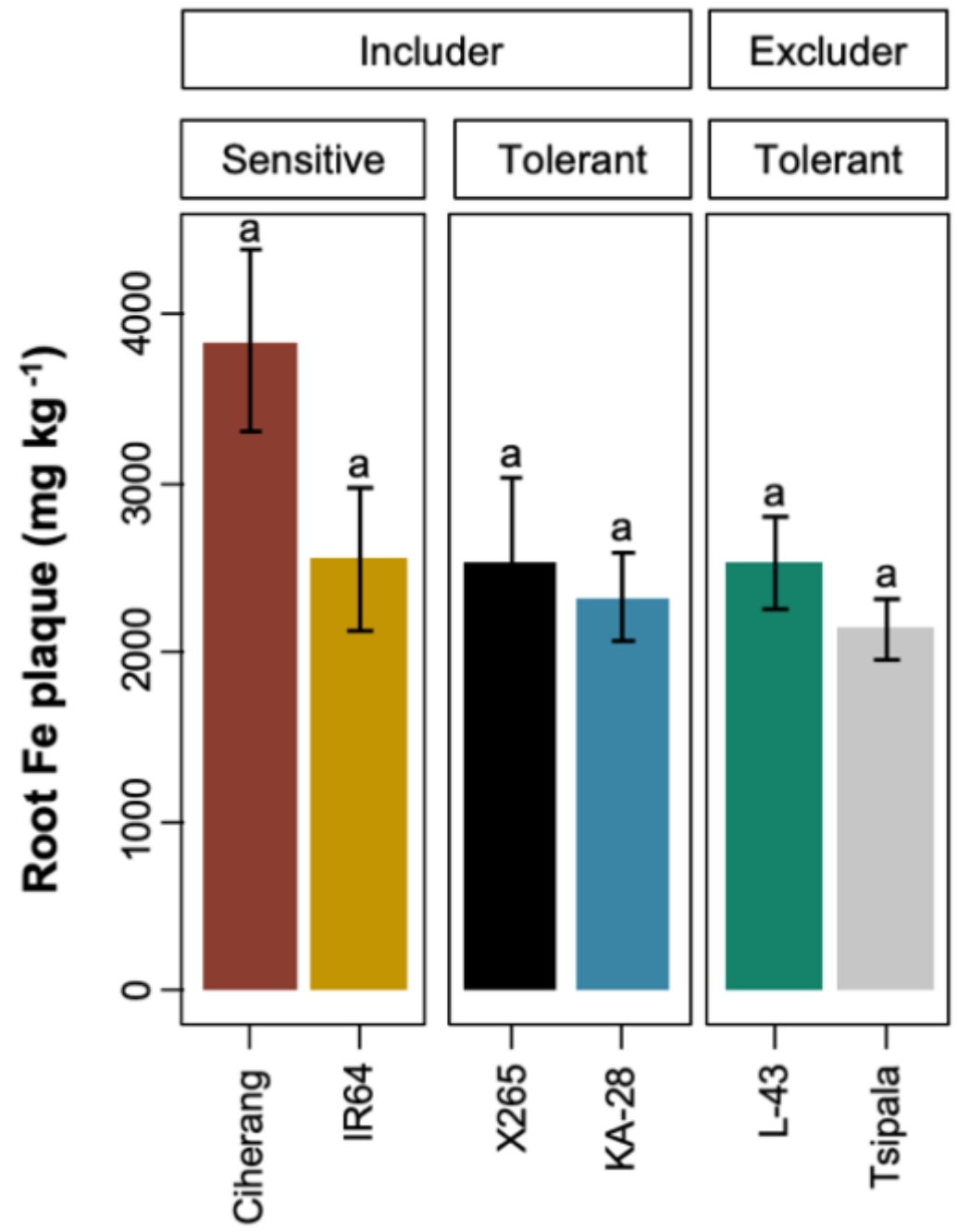

Fig. S4: Fe concentration at the booting stage in root plaque.

The experiment was conducted during 2023–24 wet season with a similar experimental setup. Data are means ± standard errors (n = 4). Common letters indicate no significant difference in Tukey's HSD test.

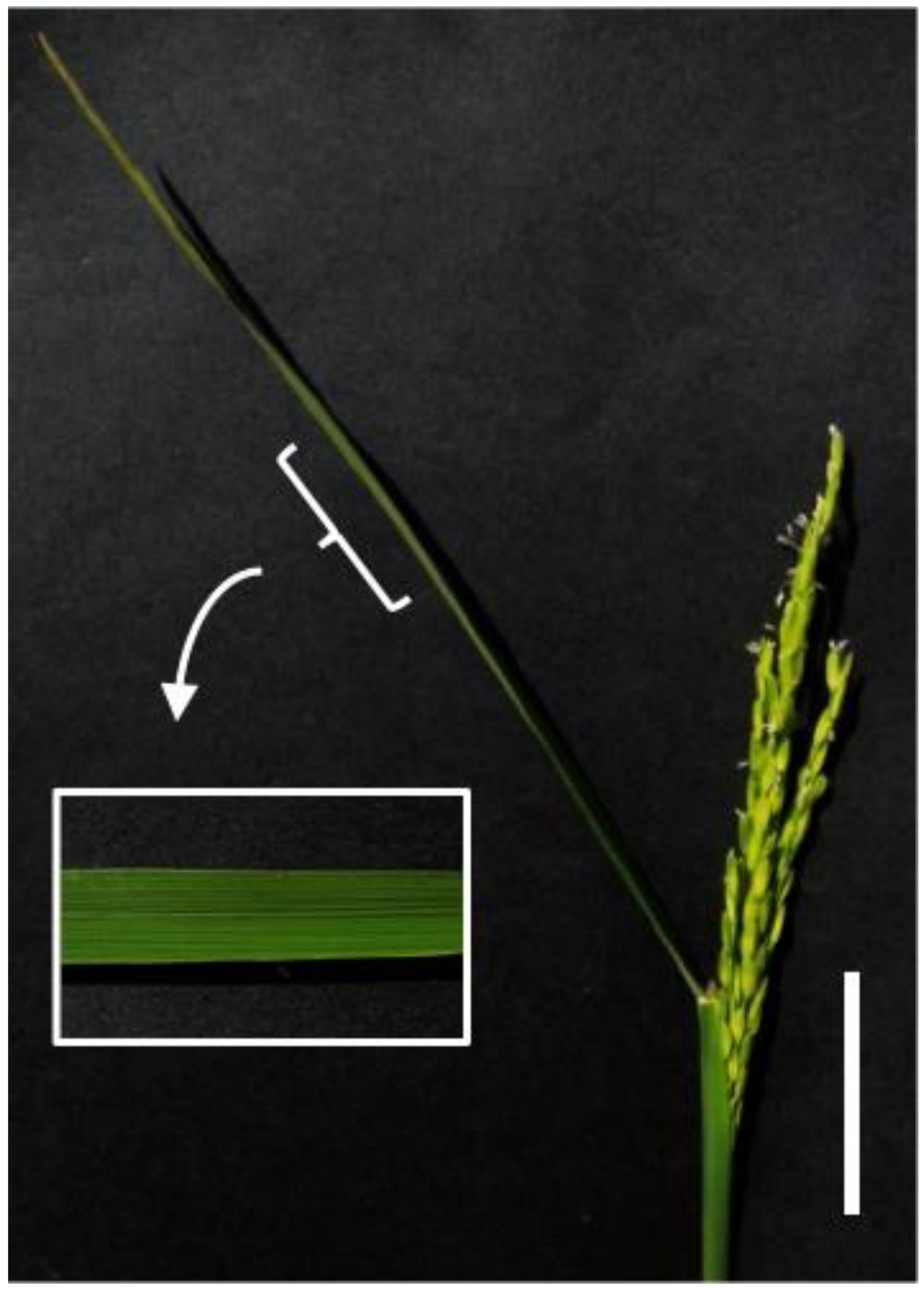

Fig. S5: Appearance of samples used for gene expression analysis in Year 3.

The middle part of flag leaf indicated by a white bracket was used for the expression analysis. Scale bar = 5 cm.

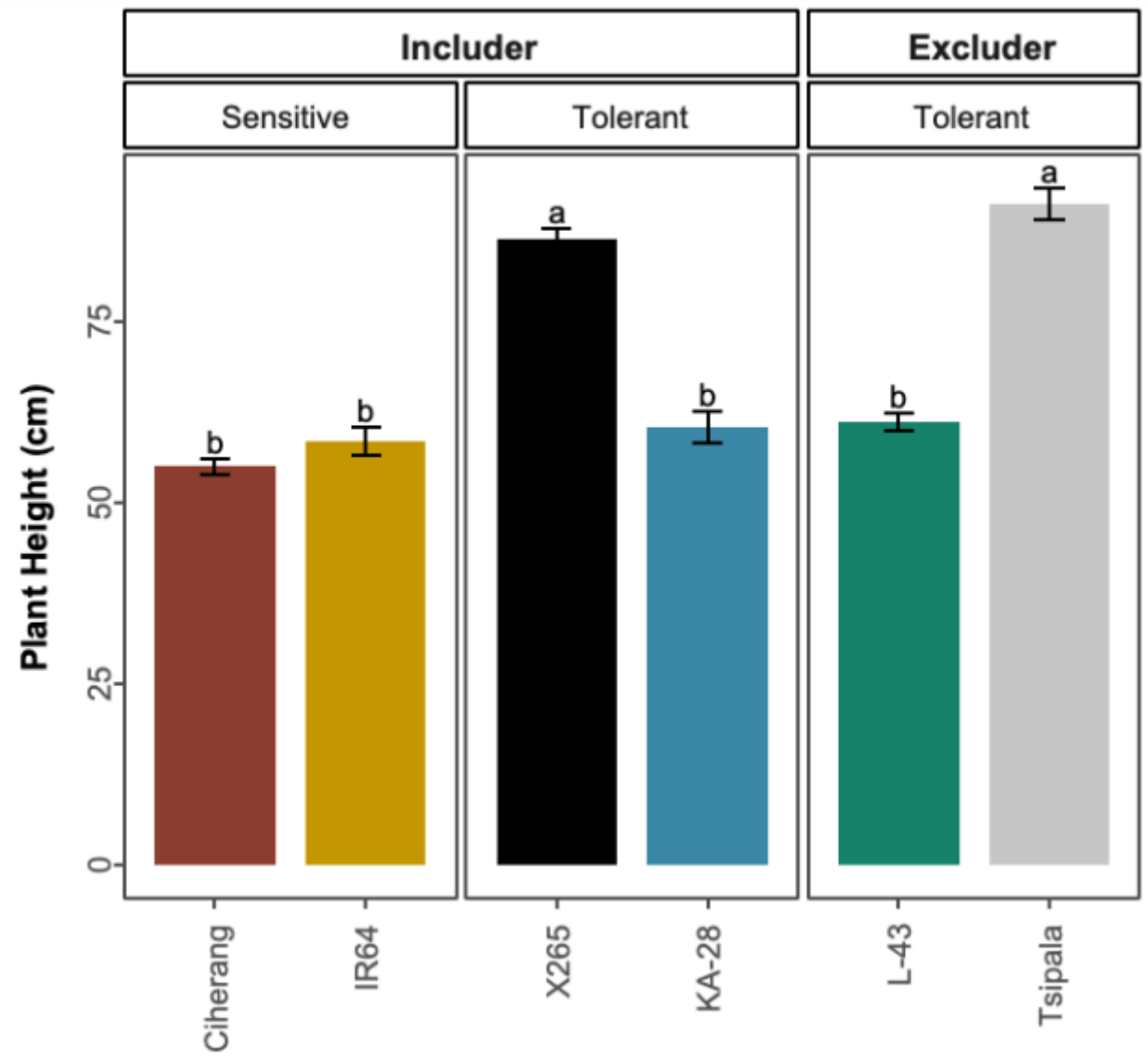

Fig. S6: Plant height at maturity across genotypes from Year 1.

Data are means ± standard errors (n = 12). Common letters indicate no significant difference in Tukey's HSD test.

Table S1. List of primers used in the study

Gene name	Gene ID	5'-sequence-3'
OsC3H38	Os05g0564200	CACAACAGGCCAACTGTGTC
		GAGGGTCTCAACCTCACCAA
OsVIT2	Os09g0396900	GCCGTGCAGTCCATCTAAGT
		TACACTACAAGCACGCAGCA
OsMGT1	Os01g0869200	TAGGGATGAGCTGGAGCACT
		TTTCACGGCCACTCTCTGTC
OsNIR	Os01g0357100	ACGACAATATGCCTTCGCCC
		AGAATGAACACAACTTTTGGGCT

Supplementary Note S1 | Effect of Fe^{2+} oxidation on cation exchange

Theory (after Kirk, 2004)

Oxidation of exchangeable Fe^{2+} ions in the rhizosphere produces ferric hydroxide and H^+: $4Fe^{2+} + O_2 + 10H_2O = 4Fe(OH)_3 + 8H^+$. The resulting decrease in Fe^{2+} concentration in the soil exchange complex, accumulation of insoluble ferric hydroxide and decrease in pH together cause changes in the distributions of other cations between the exchange complex and soil solution. Cations will be sorbed on the newly precipitated $Fe(OH)_3$ as well as on the original soil exchange sites. But for simplicity, we consider the cation exchange properties of the soil to be constant.

Representing monovalent cations as M^+ and divalent cations as M^{2+}, for a simple model we have six unknown variables: the concentrations of M^+, M^{2+} and Fe^{2+} in the soil solution and on the exchange complex. We therefore need six equations. We use (a) for monovalent-divalent exchange:

$$\frac{[\mathrm{M}^+]_\mathrm{L}}{\sqrt{[\mathrm{M}^{2+}]_\mathrm{L} + [\mathrm{Fe}^{2+}]_\mathrm{L}}} = K_{\mathrm{E1}} \frac{[\mathrm{M}^+]_\mathrm{E}}{[\mathrm{M}^{2+}]_\mathrm{E} + [\mathrm{Fe}^{2+}]_\mathrm{E}} \quad (1)$$

where subscripts L and E indicate the soil solution and exchange complex, and K_{E1} is an exchange coefficient (this is the Gapon equation, commonly used for monovalent-divalent exchange in soil); (b) for divalent-divalent exchange:

$$\frac{[\mathrm{M}^{2+}]_\mathrm{L}}{[\mathrm{Fe}^{2+}]_\mathrm{L}} = K_{\mathrm{E2}} \frac{[\mathrm{M}^{2+}]_\mathrm{E}}{[\mathrm{Fe}^{2+}]_\mathrm{E}} \quad (2)$$

where K_{E2} is another exchange coefficient; (c) for electrical neutrality in the soil solution:

$$2[\mathrm{Fe}^{2+}]_\mathrm{L} + 2[\mathrm{M}^{2+}]_\mathrm{L} + [\mathrm{M}^+]_\mathrm{L} + [\mathrm{H}^+]_\mathrm{L} = [\mathrm{Anions}]_\mathrm{L} + [\mathrm{HCO_3}^-]_\mathrm{L} \quad (3)$$

where Anions represents non-carbonate anions (e.g. Cl^-, NO_3^-); and (d) three equations for the mass balances of the three cations in the exchange complex and solution.

The value of $[HCO_3^-]_L$ is found from the pH and CO_2 pressure, P_{CO_2}:

$$[\mathrm{HCO_3}^-]_\mathrm{L} = K_\mathrm{C} K_\mathrm{S} P_{\mathrm{CO_2}} / 10^{-\mathrm{pH}} \quad (4)$$

where K_C is the apparent first dissociation constant of H_2CO_3 and K_S is the solubility of CO_2 in water. The change in pH due to Fe^{2+} oxidation depends on the soil pH buffer power, b_{HS} = –d[acid]/dpH. In most soils, b_{HS} is constant over a pH range of 1 or 2 units near neutral pH. Hence, from the stoichiometry of the oxidation reaction,

$$\Delta\mathrm{pH} = 2\Delta[\mathrm{Fe}^{2+}]/b_{\mathrm{HS}} \quad (5)$$

Parameter values

The concentration of oxidizable Fe^{2+}, CO_2 pressure, pH and pH buffer power of the reduced soil were measured as described in Materials and methods. The value of $[M^+] + [M^{2+}]$ was set equal to the measured cation exchange capacity (CEC) of the air-dry soil. The ratio $[M^+] / [M^{2+}]$ was set to give $[M^+]_L / [M^{2+}]_L$ reflecting the observed relative rates of uptake of K^+ and Ca^{2+}. For simplicity we set $K_{E1} = 1$ and $K_{E2} = 1$. The value of [Anions] was given a realistic value for a low fertility Aquult. The ratio of soil solution to solid was given a typical value for a flooded rice soil

Predicted cation exchange during Fe^{2+} oxidation

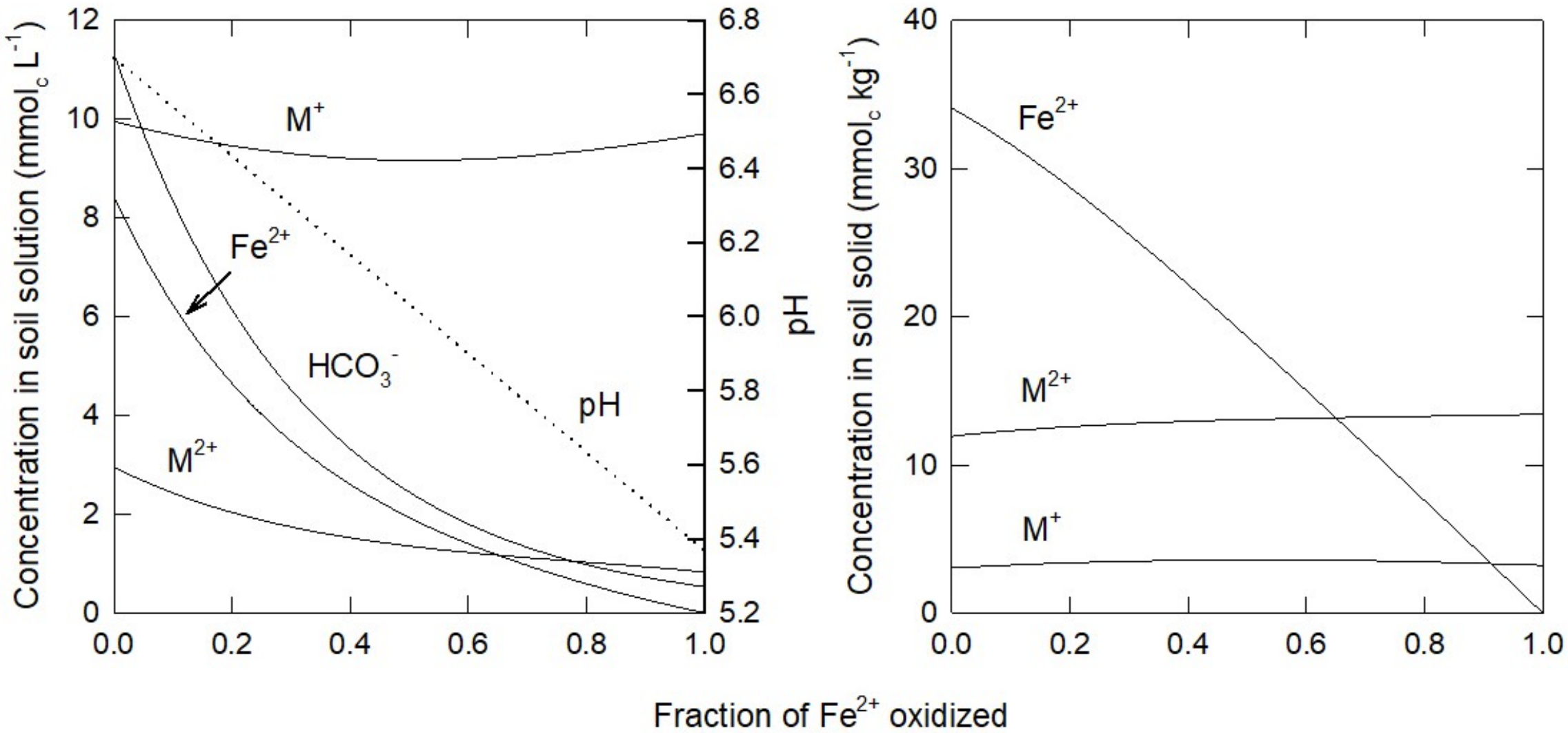

Figure 1 Calculated changes in the composition of soil solution (left hand panel) and soil solid (right hand panel) as Fe^{2+} is oxidized. Note the concentrations are in terms of moles of charge (mol_c). Parameter values: CEC of air-dry soil = 24 $mmol_c$ kg^{-1}, $[Fe^{2+}]$ = 20 mmol kg^{-1}, $[M^+]$ = 10 mmol kg^{-1}, $[M^{2+}]$ = 7 mmol kg^{-1}, $[Anions]_L$ = 10 $mmol_c$ L^{-1}, initial pH = 6.7, P_{CO_2} = 15 kPa, b_{HS} = 30 mmol kg^{-1} pH^{-1}, K_{E1} = 1, K_{E2} = 1, ratio of soil solution to solid = 0.7 L kg^{-1}.

The following points are evident from Figure 1.

1. In the initial soil before Fe^{2+} oxidation, the order of total concentrations (in terms of moles of charge) is $M^+ < M^{2+} << Fe^{2+}$ whereas the order of concentrations in solution is $M^{2+} < Fe^{2+} < M^+$ (3.0, 8.4 and 10.0 $mmol_c$ L^{-1}, respectively). This reflects preferential sorption of the divalent M^{2+} and Fe^{2+} on soil exchange sites compared with monovalent M^+, as represented by the square root term in Equation (1).
2. As Fe^{2+} is oxidized, exchangeable Fe^{2+} decreases but exchangeable M^+ is maintained and exchangeable M^{2+} slightly increases; simultaneously the pH decrease causing HCO_3^- to decrease and so the overall strength of the soil solution (= $[HCO_3^-]_L$ + $[Anions]_L$) decreases. As a result, M^{2+} in solution decreases to less than a third of its initial value but M^+ is maintained, again reflecting preferential sorption.
3. The relative decrease in M^{2+} in solution is smaller than the decrease in Fe^{2+}.

These effects will be more marked for micronutrient cations with low concentrations in plant-accessible forms in the soil (e.g. Mn^{2+} and Zn^{2+} in this soil) because they are strongly sorbed in specific adsorption reactions to a greater extent than the macronutrient cations, which have much higher concentrations. In the limit, the rate of ion uptake into a root is proportional to the concentration in solution at the root surface. Hence these calculations indicate why Fe^{2+} oxidation negatively affects uptake of divalent cations but has a smaller effect on monovalents.

Reference

Kirk GJD (2004) The biogeochemistry of submerged soils. Section 3.7.5. Wiley, Chichester. DOI:10.1002/047086303X
